# Chronotype characteristics of professional athletes in China: a comprehensive descriptive study

**DOI:** 10.1186/s40101-023-00343-2

**Published:** 2023-11-08

**Authors:** Chenhao Tan, Jiaojiao Lu, Jinhao Wang, Yan An, Guohuan Cao, Defeng Zhao, Jun Qiu

**Affiliations:** Shanghai Research Institute of Sports Science (Shanghai Anti-Doping Agency), Shanghai, 200030 China

**Keywords:** Chronotype, Athlete, Professional, ASSQ, Mental energy

## Abstract

**Background:**

Chronotype has gained recognition as a significant factor in enhancing athletic performance. This study aimed to deepen our understanding of athletes’ sleep chronotypes and provide a foundation for developing evidence-based training and competition programs. By comprehensively describing and analyzing the chronotype characteristics of Chinese professional athletes, considering individual and sports factors, sleep quality and habits, and mental energy, this research aimed to contribute valuable insights to the field.

**Methods:**

A sample of 1069 professional athletes from sports teams in Shanghai completed the Athlete Sleep Screening Questionnaire and the Athlete Mental Energy Scale to assess chronotype, sleep quality, sleep-influencing habits, and mental energy.

**Results:**

Among athletes, sleep typology fell within the intermediate range, slightly leaning toward morningness. Male athletes and those who engaged in static sports displayed a greater propensity for morningness. Age correlated with a preference for eveningness. High-level athletes exhibited a stronger inclination toward eveningness. Sleep quality issues were associated with an inclination toward eveningness. Daily caffeine intake and the habit of using electronic devices before bedtime are also linked to eveningness. Chronotype demonstrated the ability to predict various dimensions of athletes’ mental energy. It was the strongest predictor of vigor, but the loadings were smaller than those of sleep quality.

**Conclusion:**

Chinese athletes’ chronotypes primarily exhibit distinct characteristics related to individual factors such as gender, sports discipline, and ranking, as well as habits like caffeine consumption and electronic device use. Moreover, these sleep patterns demonstrate predictive capabilities across all dimensions of athletes’ mental energy. This study sheds light on Chinese athletes’ unique sleep chronotype attributes, enriching our understanding of sleep patterns among professional athletes under various systems. These insights offer an initial basis for enhancing the effectiveness of athlete scheduling and training management.

## Background

Sleep plays a crucial role in ensuring athletes’ health and significantly impacts their athletic performance [1]. Adequate sleep can enhance athletes’ physiological and psychological functions, improve fatigue recovery, and even reduce the risk of sports injuries [1–5]. Athletes’ sleep, as a natural means of achieving optimal competitive states, has garnered increasing attention.

Chronotype refers to an individual’s preference for being an early riser or a night owl, reflecting their inclination toward specific circadian rhythms [6, 7]. Similar to the extensive research on sleep quality, studies investigating chronotype have consistently unveiled significant correlations with various facets of athletic performance, encompassing physiological, psychological, and sport-specific skill sets [8–10]. In light of these discoveries, researchers have proposed chronotype-related strategies to enhance training and competition outcomes [7, 11–13]. Indeed, chronotype has emerged as an innovative approach to elevate training and competitive performance [14].

However, this pioneering approach has garnered insufficient attention, particularly among Chinese athletes, where research remains notably scarce. Investigations into athlete chronotypes are predominantly confined to athletes hailing from European, American, and Oceanic nations [15–19]. These studies bear significant potential in elucidating the characteristics and implications of chronotype within the professional athlete community. Nevertheless, it is imperative to acknowledge that while chronotype indeed harbors intrinsic stability and biological underpinnings [20, 21], social factors such as dietary habits, living conditions, and social interactions also influence the formation of chronotype [22]. The impact of these social determinants is indeed discernible among athletes.

In East Asia, researchers in Japan and Korea have delved into the chronotype of their elite athletes, revealing that most surveyed Japanese athletes manifest intermediate chronotypes, while most Korean athletes tend toward eveningness [8, 23]. This contrasts with findings from some studies suggesting a higher prevalence of morningness among elite athletes [15, 17], underscoring the potential sway of social factors.

Thus, can the attributes of chronotype among Chinese professional athletes, who share similar ethnic, regional, and cultural backgrounds with these studies, be directly extrapolated to delineate and inform practice? In reality, such an assertion oversimplifies the matter. Due to the distinctive facets of China’s athletic training and management system [24], Chinese professional athletes are ensconced within a unique institutional and environmental framework that markedly diverges from numerous other nations. The peculiarities of this system encompass regulatory aspects and environmental conditions, which may wield a specific influence on their chronotype.

In competitive sports, athletes in China’s highly centralized whole-nation system (nationwide system) grapple with a distinct training and living milieu [24–26]. Within the nationwide system, sports training receives financial backing from governmental bodies, with exceptionally gifted athletes embarking on rigorous collective training as early as middle school, progressing into professional athletes within a structured team-oriented environment. Some athletes may commence this journey even during their elementary school years. While these athletes relish economic security through government sponsorship, covering their daily livelihood, training expenses, and medical needs, they encounter stringent institutional regulations, often contending with selection pressures [26, 27]. Even for athletes still of school age, training takes precedence over their daily routines [26]. This system markedly deviates from the more prevalent focus on student-athletes in many studies [28]. The specific characteristics stemming from this institutional framework and cultural context have yet to be explored concerning the chronotype among athletes.

This study centers on professional athletes in China, utilizing specialized measurement tools developed explicitly for athletes to delineate and assess their chronotypes. The primary objective is to offer a comprehensive overview of chronotype characteristics among Chinese professional athletes, enriching existing research and advancing our comprehension of athlete chronotypes. Moreover, in conjunction with previous studies, conducting cross-cultural and cross-institutional comparisons of chronotypes can facilitate a more profound analysis of the potential impact of the management systems within which Chinese athletes operate. This research is expected to be a valuable reference for subsequent investigations and practical implementations in athlete sleep research and management.

Specifically, this study will sequentially focus on three aspects of chronotype among Chinese athletes. Firstly, it examined athletes’ demographic and sports factors commonly explored in previous research, including demographic variables, sports types, and levels of expertise. To capture the impact of sleep on cognitive abilities, this study categorized sports based on their cognitive demands [29], going beyond the traditional dichotomy of individual versus team sports. This approach facilitated a more in-depth analysis of the relationships between sleep and sport-specific characteristics. Secondly, the study investigated sleep symptoms and habits of athletes, employing a specialized sleep scale for athletes that encompassed dimensions related to athlete sleep quality, specific sleep disorders, and lifestyle habits that influence sleep. Finally, the study examined factors influencing athletic performance. As the study encompassed various sports, the physiological responses and other performance-related factors differed [9]. Therefore, the focus was on psychological factors that are relevant across different sports. Specifically, the study analyzed the relationship between chronotype and mental energy from a training needs perspective. Mental energy is the ability to sustain prolonged thinking, maintain attention, and block distractions to achieve success [30]. Unlike specific physiological functions or performance metrics that vary across sports, mental energy can support performance in all sports without being influenced by inconsistencies in performance indicators.

Furthermore, as a specialized sleep questionnaire designed for athletes was utilized, this study also conducted an additional assessment of the tool’s convergent validity. This step was undertaken to augment the reliability of the obtained results.

## Methods

### Participants

One-hundred four Chinese professional athletes (sample 1) were recruited to conduct a supplementary assessment of the validity of the chronotype subscale of the ASSQ among athletes. This group consisted of 49 males and 55 females, with an average age of 18.63 years (*SD* = 3.39).

The formal sample used to analyze chronotype comprised 1069 athletes from various professional sports teams in Shanghai (sample 2). All athletes participated in this study voluntarily. This research constituted a part of an extensive psychological assessment encompassing all professional athletes under the management of the Shanghai sports administrative system. With only a few exceptions attributable to competitions or off-site training, most professional athletes engaged in long-term training at training centers or specialized institutions took part in this study. Consequently, the athletes included in this research are highly representative.

Among them, 136 athletes were excluded due to the following criteria: not undergoing long-term training at athletic training bases, having coaching duties, training with their current team for less than one year, or being under 14 years old. The final sample consisted of 933 athletes, comprising 470 males and 463 females. The mean age of the participants was 19.47 years (*SD* = 3.96), and their average training experience in their current sport was 8.33 years (*SD* = 4.05). The age distribution reflected the typical range among Chinese professional athletes, encompassing various career stages, from emerging talents to seasoned performers. Among the participants, 383 athletes held the title of “national athlete” or higher, 314 were classified as “first-class athletes,” and 236 were categorized as “second-class athletes” or lower. These three designations were used to categorize the athletes into high (highly nationally/moderately internationally competitive), middle (highly provincially/moderately nationally competitive), and low (provincially competitive) levels of professional athletes. The data used in this study were collected through a large-scale survey conducted among athletes from Shanghai’s province-level sports teams. Some athletes had national team experience.

All participants underwent long-term training at athletic training bases managed by Shanghai’s sports administration. Their sports disciplines included football, basketball, volleyball, track and field, handball, water polo, swimming, modern pentathlon, fencing, table tennis, badminton, boxing, judo, taekwondo, martial arts, gymnastics, and field hockey. Based on previous categorizations of cognitive demands [29], the athletes were classified into static sports (*n* = 340), interceptive sports (*n* = 180), and strategic sports (*n* = 380). Thirty-three athletes could not be clearly classified and were excluded from analyses involving sports types. Static sports referred to individual sports such as athletics and swimming, interceptive sports involved individual opponents like fencing, table tennis, and badminton, while strategic sports encompassed team sports.

The study was approved by the Ethics Committee of the Shanghai Research Institute of Sports Science (Shanghai Anti-Doping Agency), no. LLSC20220005. Participants provided written informed consent before participating in the study, and permission was obtained in advance from their respective sports teams (for minors, consent was provided by their guardians).

### Scales

#### Athlete Sleep Screening Questionnaire (ASSQ)

The Athlete Sleep Screening Questionnaire (ASSQ) is a specialized tool designed for screening sleep quality among athletes, with athletes responding based on their recent sleep conditions [31]. These responses guide subsequent assessments and intervention recommendations for their sleep.

Initially, the ASSQ comprised 15 items, primarily sourced from various self-report sleep scales. Experts categorized these items into sections related to total sleep time, insomnia, sleep quality, chronotype, travel disturbance, and sleep disordered breathing, guided by clinical significance [31]. Subsequent studies assessing the reliability and validity of the ASSQ led to the development of a 16-item version. This revised version includes sections on sleep difficulty score, modifiers, and other items of interest and has been incorporated into the Sport Mental Health Assessment Tool 1 [32, 33]. For this study, we utilized this validated version of the ASSQ [32].

The sleep difficulty score (SDS) consists of 5 questions (items 1, 3–6), with higher scores indicating a more pronounced degree of sleep difficulty. The ASSQ’s modifiers encompass indicators for sleep breathing disorders, travel-related issues, and chronotype [31, 32]. The sleep breathing disorders indicator comprises two questions (items 13 & 14), with an affirmative response to either indicating the presence of this problem. The travel-related indicator contains two questions: a positive response to one (item 11) suggests the need for targeted sleep education, while a positive response to the other (item 12) suggests the need for medical or sleep science diagnosis and intervention. The chronotype section of the ASSQ comprises four questions (items 7–10). The Athlete Morningness/Eveningness Scale was used in the initial item pool. However, it was subsequently replaced by the composite scale of morningness during the compilation process, which continued to the final version. Higher scores in the chronotype dimension indicate more pronounced “morningness” characteristics (conversely, “eveningness”), allowing for interventions such as light therapy or melatonin supplementation based on the scores [32]. Other items of interest pertain to issues related to napping, caffeine intake, and pre-sleep electronic device usage, with higher scores indicating greater frequency of these behaviors.

This scale has been applied in studies involving athlete populations and has been introduced in China with the development of a Chinese version [33–36]. The Chinese version of the ASSQ’s SDS comprises four items (with the sleep medication item removed) with scores ranging from 0 to 17, while the chronotype section includes three items with scores ranging from 0 to 11. Among the other items of interest, “napping” has been explicitly defined as “afternoon nap” to align with the habits of Chinese athletes. It should be noted that in the Chinese version, the validity result of the chronotype subscale was provided based on the Pittsburgh Sleep Quality Index [36]. However, the validity between the chronotype subscale and other chronotype scales was not provided. Therefore, we used the Reduced Morningness-Eveningness Questionnaire in this study to provide additional convergent validation information.

This study employed the ASSQ’s chronotype subscale to assess athletes’ chronotypes. Continuous scores were included in the analysis to better describe the degree of morningness or eveningness, following a precedent from prior research [10]. The SDS was used to represent overall sleep quality. Responses on sleep breathing disorders, travel sleep issues, afternoon napping, caffeine intake, and pre-sleep electronic device usage were used to describe athletes’ sleep disorders and habits. Responses to these questions were recorded. Specifically, (1) sleep breathing disorder results were categorized into “present” or “absent,” with an affirmative response to either item coded as “present”; (2) travel-related sleep issues were categorized into “absent” or “present,” with an affirmative response to either item coded as “present”; (3) afternoon napping was recoded into “almost every training day” (5–7 days per week) and “not every training day” (other options); (4) caffeine intake was recoded into “almost none” (less than one can per day) and “intake” (other options); and (5) pre-sleep electronic device usage was recoded into “every training day” (daily) and “not every training day” (other options).

### Athlete Mental Energy Scale (AMES)

The AMES measures the intensity of motivation, confidence, attention, and emotions in athletes using 18 items rated on a 1–6 scale. It comprises six subscales: vigor, confidence, motivation, tireless, concentration, and calm [37]. Each dimension of the AMES consists of three items, with scores ranging from 3 to 18. The Chinese version of the AMES has demonstrated strong reliability and validity, with adapted versions in other Asian languages also displaying good cross-cultural validity [38]. The simplified Chinese version provided by the original authors maintains the same structure and exhibits good psychometric properties. The AMES has been effectively utilized in athlete research [39–41].

### Reduced Morningness-Eveningness Questionnaire (rMEQ)

The convergent validity of the chronotype subscale of ASSQ was assessed using the rMEQ. The rMEQ consists of five items that evaluate morningness and eveningness tendencies, with scores ranging from 4 to 25. Higher scores indicate a stronger morningness tendency. The Chinese version of the rMEQ has demonstrated good reliability and validity [42].

### Procedure

All measures were completed individually using mobile phones, with scheduling based on competition and training demands. Specifically, the 1-month period before and after testing avoided significant domestic and international competitions and off-site training, allowing athletes to maintain their regular lifestyle and training routines. In validating the chronotype subscale, athletes completed the paper-based questionnaire or an online survey at the laboratory site.

### Statistical analysis

Statistical analysis was conducted using SPSS 26.0. At first, the normality of the two variables in sample 1 was examined. The normality of rMEQ was confirmed (Shapiro–Wilk = 0.978, *p* = 0.079), while the chronotype variable of ASSQ did not pass a strict normality test (Shapiro–Wilk = 0.919, *p* < 0.001). Therefore, Spearman’s correlation analysis was used to analyze validity. For sample 2, recoding was performed for factors such as sleep breathing disorder, travel sleep issues, napping, caffeine intake, and pre-sleep electronic device usage. Since the chronotype score did not pass a strict normality test (Shapiro–Wilk = 0.946, *p* < 0.001), even though they exhibited approximate normality in distribution, nonparametric tests, specifically Wilcoxon signed-rank tests, Mann–Whitney *U*-tests, and Kruskal–Wallis ANOVA, were used to ensure the robustness of the results. Cliff’s δ was used as the effect size in pairwise comparisons, and Bonferroni correction was applied for multiple comparisons. Additionally, Spearman’s correlation analysis examined the relationship between athlete age and sleep difficulty.

Considering that the level of Chinese professional athletes is not only related to their athletic skills but also highly related to their age (as higher-level rankings often require accumulated athletic achievements), a mediation model with age as a mediator variable was constructed using PROCESS v4.2 to control for the potential influence of athlete age [43]. Model 4 was employed in the analysis, with the level as the ordinal independent variable, age as the mediator variable, and chronotype as the dependent variable. A 95% confidence interval was set, and Bootstrap was set to 10,000.

Finally, a multiple linear regression was used to predict the dimensions of psychological energy with athlete chronotype as the independent variable. In this regression model, alongside chronotype, several potentially influential factors, including age, gender, and sleep quality, were incorporated using the enter method. Gender, sport type, and athletic level were set as dummy variables (with male, static type, and low-level athletes as references). *VIF* > 5 was used as the criterion for detecting significant multicollinearity. Since the regression analysis does not strictly require a specific data distribution, and data distribution has minimal impact with larger sample sizes, additional transformations or processing of the data may be overly aggressive [44, 45]. Therefore, in this study, no additional data transformation was performed based on data distribution for the statistical analysis processes involving regression analysis.

## Results

### Validation of the chronotype subscale

The analysis revealed a significant and positive correlation between the scores on the chronotype subscale of the ASSQ and the total scores on the rMEQ (*ρ* = 0.663, *p* < 0.001). This finding confirmed the convergent validity of the chronotype subscale of the ASSQ again.

### Demographic and sport factors

**Fig. 1:**
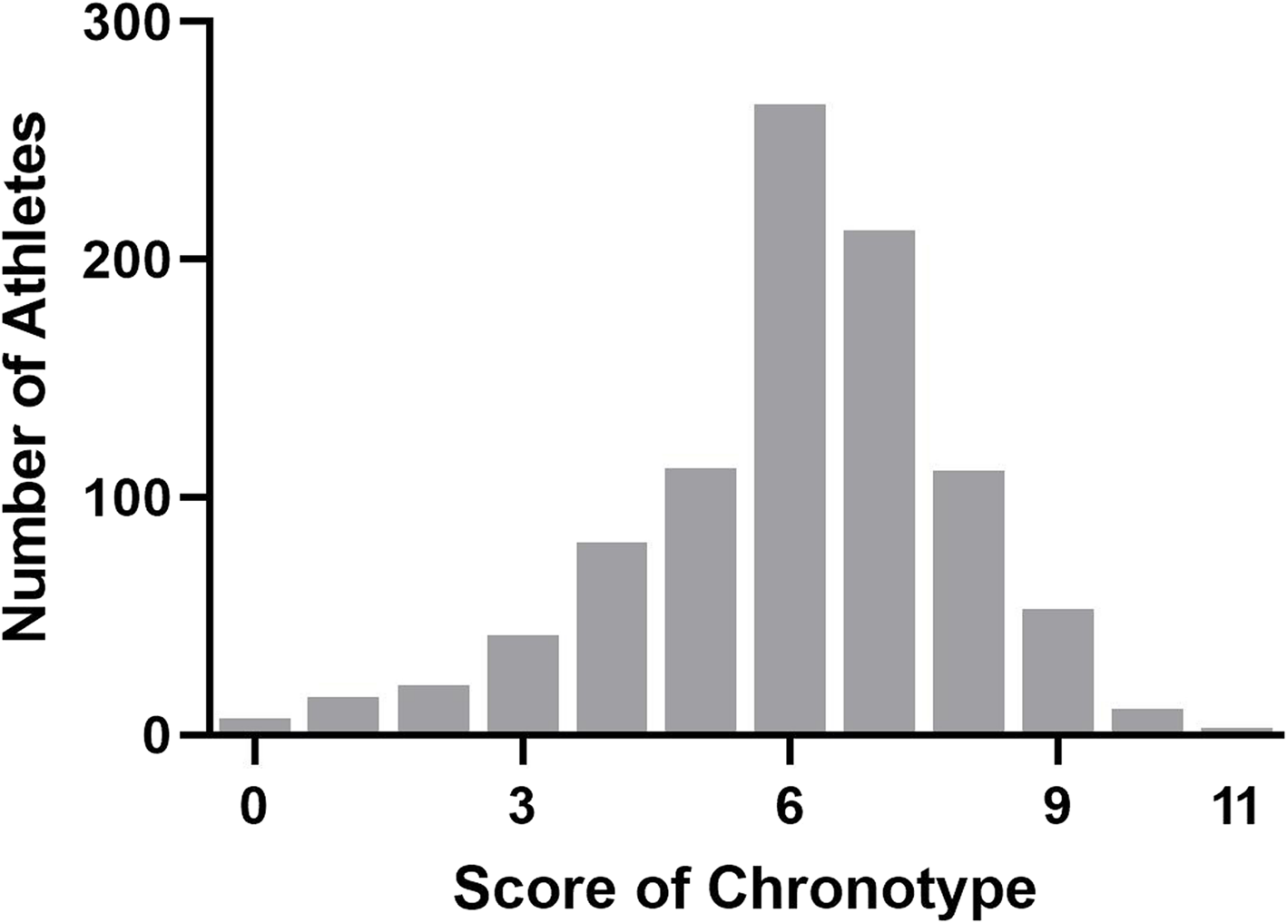
Distribution of chronotype scores among all athletes

The overall mean and distribution of scores indicate that athletes, on average, tend to exhibit a central but slightly morning-oriented chronotype (see Fig. 1, Table 1). Athletes scored significantly higher than the midpoint of the score range (0–11 points, midpoint at 5 points) on the chronotype subscale, *W* = 271,548.500 and *p* < 0.001. However, there was no significant difference between those scoring and 6 points, *W* = 119,225.00 and* p* = 0.126.

**Table 1:** Descriptive results of chronotype scores among athletes by different demographic and sports factors

		*n*	*M*	*SD*
Sex	Male	470	6.24	1.86
	Female	463	5.85	1.81
Sports	Static	340	6.28	1.91
	Interceptive	180	5.67	1.91
	Strategic	380	6.02	1.74
Level	Low	236	6.40	1.69
	Middle	314	6.33	1.69
	High	383	5.59	1.95
Total		933	6.04	1.84

Regarding gender differences, female athletes demonstrated a greater inclination toward an evening chronotype than their male counterparts. Mann–Whitney *U*-test results revealed a significant gender difference, *U* = 95,299.000, *p* < 0.001, and Cliff’s δ = 0.124.

**Fig. 2:**
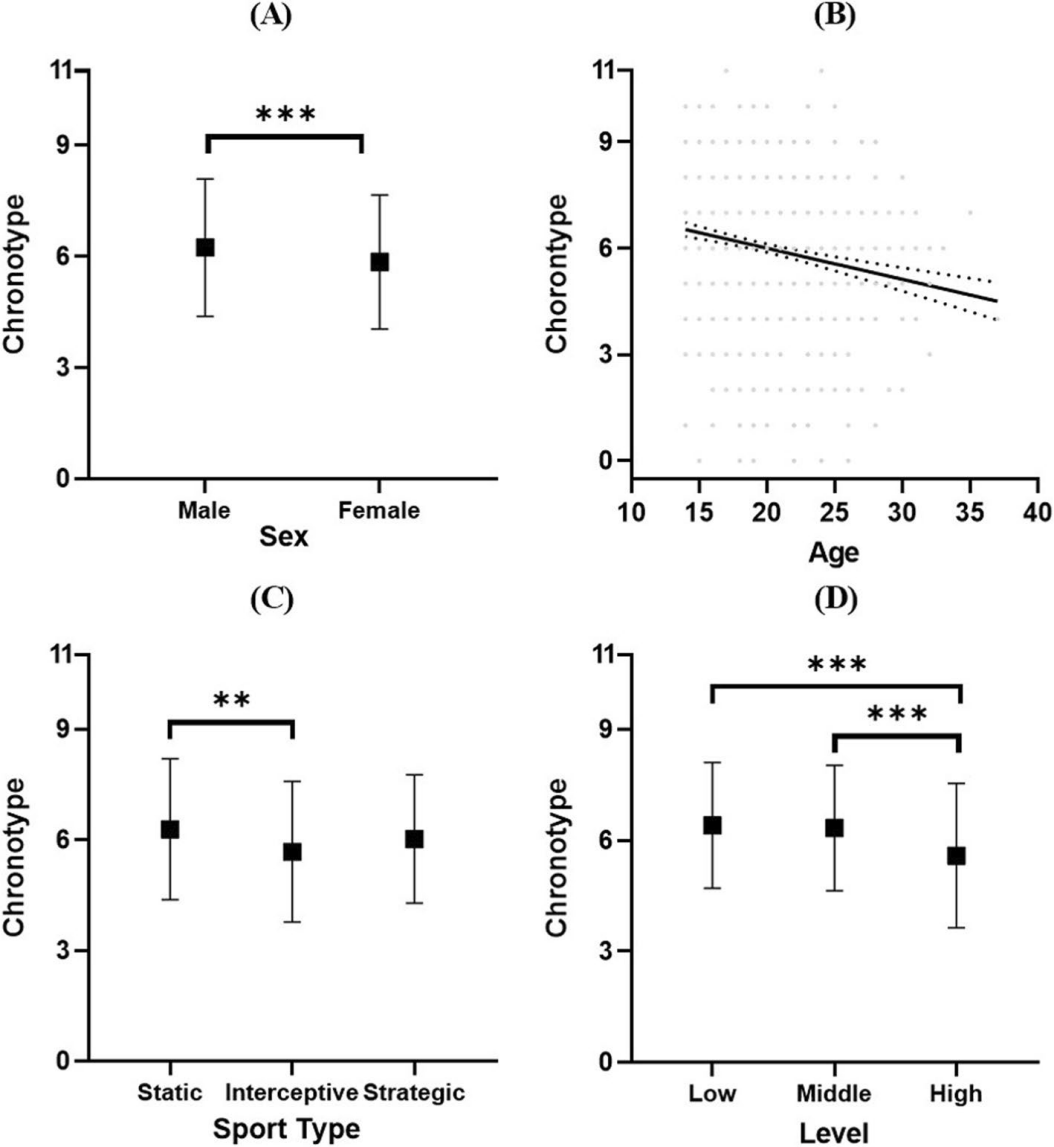
Chronotype characteristics of athletes across different demographic and sport-related factors (including differences in gender (**A**), age-related correlations (**B**), distinctions between different sports (**C**), and variations among different sports levels (**D**). *p < .050, **p < .010, ***p < .001. Dotted lines in the scatter plots represent the 95% confidence intervals for linear trends)

In terms of age, there was a significant but weak negative correlation between athlete age and chronotype scores, *ρ* =  − 0.218 and *p* < 0.001 (see Fig. 2 A–B and descriptive statistics in Table 1 for details).

Significant differences were observed among athletes from different sports, as indicated by the results of Kruskal–Wallis ANOVA, *H* = 14.215 and *p* = 0.001. Multiple comparisons revealed that athletes in static sports tended to have a more morning-oriented chronotype than those in interceptive sports, *p* = 0.001 and Cliff’s δ = 0.188. However, no significant differences were found between athletes in static sports and strategic sports, *p* = 0.057 and Cliff’s δ = 0.101. Similarly, there were no significant differences between interceptive and strategic sports athletes, *p* = 0.214 and Cliff’s δ = 0.096. Differences were also observed among athletes at different levels, *H* = 35.731 and *p* < 0.001. Multiple comparisons showed that high-level athletes were more evening oriented than low-level athletes, *p* < 0.001 and Cliff’s δ = 0.242. High-level athletes also exhibited a more evening-oriented chronotype than middle-level athletes, *p* < 0.001 and Cliff’s δ = 0.210. There were no differences between middle- and low-level athletes, *p* > 0.999 and Cliff’s δ = 0.039 (see Fig. 2 C–D).

In the analysis of mediation effects, the three levels were transformed into dummy variables, with middle- and high-level athletes serving as reference categories for low-level athletes. The results revealed an omnibus total effect of level on chronotype, with nonzero relative total effects for both levels, *F*(2, 930) = 16.463, *p* < 0.001. An omnibus direct effect was observed, with nonzero relative direct effects for both levels, *F*(2, 929) = 7.218, *p* = 0.008. Regarding relative mediation effects, when middle-level athletes were compared to low-level athletes, the relative total effect was not significant, 95% *CI* = [− 0.376, 0.234]. However, when high-level athletes were compared to low-level athletes, the relative total effect was significant, 95% *CI* = [− 1.108, − 0.522], with a direct effect of − 0.530 (95% *CI* = [− 0.888, − 0.172]) and an indirect effect of − 0.285 (95% *CI* = [− 0.501, − 0.074]), accounting for 34.98% of the total effect.

Further mediation analysis was conducted based on the differences found among sports levels, using middle-level athletes as the reference category for high-level athletes. The results indicated that in terms of relative mediation effects, when high-level athletes were compared to middle-level athletes, the relative total effect was significant, 95% *CI* = [− 1.013, − 0.474], with a direct effect of − 0.560 (95% *CI* = [− 0.860, − 0.259]) and an indirect effect of − 0.184 (95% *CI* = [− 0.327, − 0.047]), accounting for 24.77% of the total effect.

### Sleep symptoms and habits

**Fig. 3:**
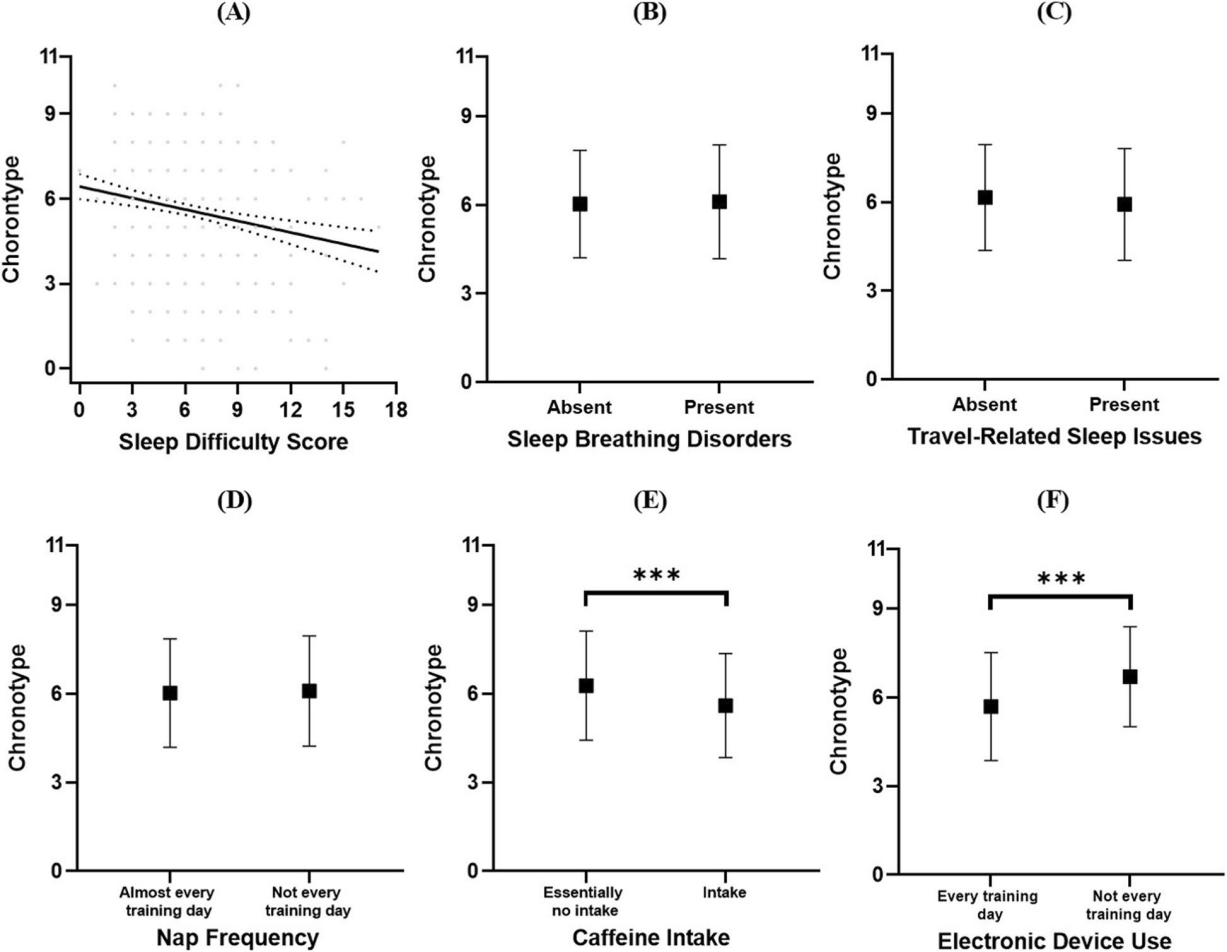
Chronotype characteristics of athletes across different sleep symptoms and habits (including correlations with the severity of sleep disorders (**A**), differences in the presence of sleep breathing disorders (**B**), differences in the presence of travel-related sleep issues (**C**), differences in napping habits (**D**), differences in daily caffeine intake (**E**), and differences in daily pre-sleep electronic device usage (**F**). *p < .050, **p < .010, ***p < .001. Dotted lines in the scatter plots represent the 95% confidence intervals for linear trends)

There was a relatively low negative correlation between sleep difficulties and chronotype, *ρ* =  − 0.196, *p* < 0.001. Athletes with apparent sleep breathing disorders showed no significant differences in chronotype (*U* = 0.115, *p* = 0.735, Cliff’s δ =  − 0.015). Similarly, athletes experiencing travel-related sleep issues exhibited no significant differences in chronotype (*U* = 101,044.500, *p* = 0.057, Cliff’s δ = 0.070) (see Table 2 and Fig. 3A–C).

**Table 2:** Descriptive results of chronotype scores among athletes by different sleep symptoms and habits

		*n*	*M*	*SD*
Sleep breathing disorders	Absent	721	6.03	1.82
	Present	212	6.10	1.92
Travel-related sleep issues	Absent	481	6.16	1.79
	Present	452	5.92	1.89
Nap frequency	Almost every training day	620	6.02	1.83
	Not every training day^a^	313	6.09	1.87
Caffeine intake	Essentially no intake	619	6.27	1.84
	Intake^b^	314	5.60	1.76
Electronic device use	Every training day	604	5.69	1.82
	Not every training day^c^	329	6.70	1.69

Note: ^a^Including “three or four times per week” (*n* = 236), “once or twice per week” (*n* = 51), and “none” (*n* = 26). ^b^Including “1–2 cans per day” (*n* = 269), “3 cans per day”(*n* = 35), “ 4 cans per day” (*n* = 7), and “5 cans per day” (*n* = 3). ^c^Including “not at all (*n* = 78),” “1 − 3 times per week” (*n* = 150), and “4 − 6 times per week” (*n* = 101)

Regarding sleep habits, athletes who napped daily showed no significant differences in sleep type (*U* = 86,793.000, *p* = 0.545, Cliff’s δ =  − 0.019). Athletes who consumed caffeine-containing foods daily exhibited differences in sleep type (*U* = 75,481.000, *p* < 0.001, Cliff’s δ =  − 0.223). Athletes who did not consume caffeine tended to be more morning oriented. Athletes who used electronic devices daily before sleep showed differences in sleep type (*U* = 66,589.000, *p* < 0.001, Cliff’s δ =  − 0.330). Athletes with a daily pre-sleep electronic device usage tendency leaned toward eveningness (see Table 2 and Fig. 3D–F).

### Mental energy

Chronotype predicted dimensions of mental energy, although the model fit was not optimal enough. After controlling for age, gender, and sleep quality, chronotype still predicted all dimensions, with stronger effects on vigor. However, sleep difficulties may have been a stronger predictor than chronotype, and the model fit remained suboptimal (see descriptive results, standardized coefficients, and fit indices in Tables 3 and 4).

**Table 3:** Descriptive results of mental energy

	*M*	*SD*
Vigor	12.26	2.86
Confidence	11.71	2.96
Motivation	13.60	3.04
Concentration	12.28	3.38
Tireless	9.13	3.28
Calm	11.30	3.28

**Table 4:** Standardized regression coefficients and model fit of linear regression for chronotype and other factors on mental energy

		Sex	Age	Sport type		Level		SDS	Chorontype	R_adj_^2^
				Interceptive	Strategic	Middle	High			
Vigor	β	− 0.131	.089	.059	0.135	− .001	.027	− 0.334	0.176	0.193
	*p*	< .001	.019	.086	< .001	0.982	0.563	< .001	< .001	
Confidence	β	− 0.179	0.166	.037	0.126	− .007	− .003	− 0.260	0.104	0.144
	*p*	< .001	< .001	0.288	< .001	0.873	0.958	< .001	.001	
Motivation	β	− 0.109	− .021	.008	0.158	.026	.016	− 0.250	.088	0.116
	*p*	< .001	0.604	0.814	< .001	0.530	0.744	< .001	.008	
Concentration	β	− 0.101	− .028	.058	0.146	.042	.003	− 0.303	0.127	0.157
	*p*	.002	0.475	.096	< .001	0.305	0.942	< .001	< .001	
Tireless	β	− 0.133	.034	0.110	0.126	− .072	− .088	− 0.224	0.146	0.121
	*p*	< .001	0.395	.002	.001	.083	.072	< .001	< .001	
Calm	β	− 0.133	0.219	− .044	.044	.029	− .028	− 0.225	0.115	0.125
	*p*	< .001	< .001	0.220	0.232	0.488	0.562	< .001	< .001	
VIF		1.095	1.625	1.305	1.382	1.744	2.419	1.110	1.111	

## Discussion

Chinese professional athletes’ chronotypes were concentrated in the middle range but slightly skewed toward morningness. This characteristic differed from research on professional athletes in other East Asian countries. Specifically, the distribution of chronotype scores among Chinese athletes in this study was similar to that found in a study of international and national elite athletes in Japan [23], except that the latter exhibited a normal distribution. In contrast, another study of elite athletes in Korea found a higher prevalence of eveningness [8]. These differences may have reflected the overall impact of China’s administrative management of athletes’ daily lives and training schedules on chronotypes.

Chinese male athletes tended to be more morning oriented than their female counterparts. This gender-based preference contradicted the results of several large-sample surveys and meta-analyses conducted in the general population [46–48]. The differences among them may exhibit unique characteristics of professional athletes. Although no direct comparison was made, a study of South Korean athletes found a higher proportion of evening-oriented female athletes among those with a preference for eveningness [8].

Athletes’ age was correlated with an inclination toward eveningness. This phenomenon aligned with the average age results for different chronotypes among Korean athletes and conformed to the typical age-related changes in chronotype preferences [8, 49, 50]. Eveningness strengthened with age, peaking around the early 20 s [49, 50]. Since the average age of athletes in this study was around 20, representing the typical age range for Chinese administrative athletes, this age range may have coincided with increasing eveningness tendencies.

Athletes in static sports tended to be more inclined toward morningness than interceptive sports, with only a slight and nonsignificant difference from strategic sports. This result expanded upon existing research findings. Previous studies often found that individual athletes tended to be morning oriented, especially those in physically demanding sports [16, 17, 51, 52]. Static and interceptive sports in this study broadly corresponded to individual sports in previous research, while strategic sports corresponded to team sports [29]. Notably, significant differences existed among individual sports in Chinese professional athletes, while differences compared to team sports were less pronounced. The characteristics of training in each sport may have been a critical factor in this phenomenon. Different sports were believed to affect chronotype preferences due to variations in training schedules [7, 17, 53]. Under administrative oversight, Chinese professional athletes’ training times and daily routines were strictly managed [26, 27], including minimum and maximum training times. This uniform scheduling may have weakened the distinctions among sports. Differences among individual sports could have been related to physical demands and additional training time, warranting further research with training data tracking.

High-level Chinese athletes tended to be more evening oriented than lower-level athletes. This result contradicted findings from previous research [54]. More importantly, the same pattern persisted even when including age as a mediating variable. Whether this pattern had cultural factors was currently unclear. Japanese international- and national-level athletes did not exhibit a pronounced preference for eveningness [23]. However, there was no data on athletes of different levels to determine whether they were less evening oriented. Although the Korean elite athlete population exhibited clear eveningness [8], the study did not define athletic levels and lacked comparisons between different levels. Management factors might have provided an alternative explanation. Opportunities related to education and employment were associated with athletes’ achievements in China [26, 27]. High-level athletes had opportunities for further education and employment due to their achievements, which required additional efforts and may have led to a relaxation of daily management previously imposed under strict regulations. The current study did not investigate athletes’ daily activities, necessitating further research to validate this possibility.

In the realm of sleep symptoms, poor sleep quality has shown an association with a propensity toward eveningness. This discovery aligns with previous research that has illuminated the detrimental effects of chronotype on sleep quality [18]. When schedules are meticulously regulated, deviations in sleep–wake times, such as late bedtimes, can exacerbate shifts in sleep quality [55]. For Chinese athletes, personalized schedules are often unattainable, posing challenges for evening-oriented athletes. Notably, there were no discernible differences in chronotype concerning sleep breathing disorders or travel-related sleep issues. These specific sleep symptoms might involve alternative physiological mechanisms.

Regarding sleep-related habits, daily napping exhibited no correlation with chronotype. Napping is increasingly recognized as a means of enhancing training quality [56]. However, owing to the traditional Chinese cultural esteem for midday naps [57], afternoon naps have long been integrated into the daily routines of Chinese athletes under management. This study showed that most athletes habitually napped daily or on most training days, with very few athletes seldom napping. Since this study employed the ASSQ, primarily focusing on nap frequency [31], without delving into finer details like timing and duration, it may not have sufficed to elucidate the relationship between the nap characteristics of frequently napping athletes and their chronotypes. Further, in-depth research is imperative to scrutinize this relationship.

Caffeine intake was associated with a proclivity toward eveningness, notwithstanding previous studies failing to establish such a connection [58]. In this study, the majority of athletes consumed minimal caffeine. Foods with high caffeine content are not conventionally part of the Chinese diet, and dietary and anti-doping management requirements on training days might have contributed to the limited adoption of this habit. Athletes with higher caffeine consumption might do so out of functional necessity, aligning with the traits of eveningness. Coupled with the napping pattern, this might suggest the necessity for further examining lifestyles and habits across different regions and systems. This also necessitates an analysis of factors such as timing and motivation for caffeine intake [59].

The utilization of electronic devices before bedtime correlated with a predisposition toward eveningness. This discovery concurs with the prerequisites of sleep hygiene management [60]. Furthermore, it aligns with prior research findings that athletes who engage with electronic devices before bedtime typically exhibit later sleep onset [61]. The daily management of Chinese athletes seemingly had no notable impact on electronic device use, warranting the attention of management personnel.

Sleep chronotype displayed a significant but relatively weak association with various mental energy dimensions. This implies that sleep chronotype can indeed influence the athletic performance of Chinese athletes through mental energy, complementing findings related to other facets of physical performance [8, 17, 62]. However, it is imperative to note that while sleep chronotype can forecast mental energy, the role of sleep quality appears to be more paramount. This might be attributed to the influence of sleep chronotype through synchronization with daily schedules, while sleep quality directly correlates with fatigue recovery. Hence, the emphasis on chronotype should likely underscore strategies for training and competition timings [62], while sleep quality should be seamlessly integrated into routine management practices.

This study represents the first attempt to describe and preliminarily analyze the characteristics of sleep chronotype among Chinese professional athletes who operate under a nationwide system, emphasizing training and adhering to tightly regulated daily routines. Based on these findings, we can provide preliminary recommendations for Chinese athletes and those under similar management structures. First and foremost, sports organizations should pay heightened attention to the performance of female athletes, athletes engaged in interceptive sports, and high-level athletes participating in morning training or competitions. Simultaneously, they should consider other athletes’ performance when participating in nighttime competitions. In addressing sleep-related issues, medical support services should concentrate on evaluating the sleep quality of evening-oriented athletes and contemplate incorporating sleep chronotype screening when dealing with sleep-related concerns. Additionally, there is a pressing need for enhanced education on sleep hygiene, particularly concerning caffeine intake and the use of electronic devices in daily routines. Concerning athletic capabilities, it is essential to recognize the mental energy levels of evening-oriented athletes. However, it is equally vital not to oversimplify this relationship by solely attributing it to sleep chronotype and enforcing strict control over athletes’ sleep habits. Instead, it is advisable to prioritize a nuanced understanding of the distinctive features of sleep quality over time.

However, this study harbors limitations that may have constrained the research outcomes. Firstly, the study exclusively relied on self-report questionnaires tailored for athletes, thus lacking objective measures of chronotype, sleep patterns, daily routines, and athletic performance. Secondly, data collection regarding athletes’ habits and training particulars was somewhat limited, particularly concerning factors such as nap duration, timing, dietary habits, electronic device usage, and strategies for managing training fatigue and recovery. These limitations hindered a more in-depth analysis of these influential factors. Lastly, the study lacked comparative analyses across various management systems and cultures, which complicates the ability to distinguish between the effects of management systems and cultural influences.

Future research endeavors should strive to employ more objective measurement methods to validate the phenomena unearthed in this study. This includes further exploration of sleep chronotype recognition, comprehensive assessments of sleep quality, and the quantification of athletic capabilities. Additionally, future studies should seek to elucidate the underlying mechanisms driving the observed phenomena. This could be achieved by considering various factors such as daily training regimens, routines, and lifestyle habits, with particular attention to athletes’ dietary preferences and nap behaviors. Lastly, it is imperative to emphasize conducting cross-system and cross-cultural comparisons. These comparisons will facilitate a more effective delineation of the relationship between the sleep chronotype characteristics of Chinese athletes and the specific management strategies in place. This examination should encompass characteristics intrinsic to age groups, scheduling regulations, ranking systems, and habit formation. Only through such comprehensive efforts can we gain a more nuanced understanding of the intricate dynamics at play.

## Conclusion

Under China’s comprehensive sports management system, the sleep chronotype of professional athletes reveals distinctiveness primarily influenced by individual characteristics such as gender, sport types, and ranking, as well as lifestyle habits, including daytime napping and caffeine consumption. Importantly, it demonstrates the capability to predict all dimensions of athletes’ mental energy. This study provides an initial description of the manifestation and characteristics of sleep chronotype among professional athletes, operating within the unique social context of China’s competitive sports management system. These findings lay the foundation for the development of preliminary strategies to address these unique aspects. Moreover, this study underscores the necessity of considering the influence of management systems when investigating athletes’ sleep chronotypes, emphasizing the need for more in-depth research into the impact of management systems akin to that experienced by Chinese athletes.

## Data Availability

The datasets used and/or analyzed during the current study are available from the corresponding author on reasonable request.

## Abbreviations

ASSQ: Athlete Sleep Screening Questionnaire
AMES: Athlete Mental Energy Scale
SDS: Sleep Difficulty Score
ANOVA: Analysis of variance
